# Supplementary figures and images for: NEK Family Review and Correlations with Patient Survival Outcomes in Various Cancer Types
Source: Cancers (Basel). 2023 Mar 30;15(7):2067. doi: 10.3390/cancers15072067 (PMC10093199; doi:10.3390/cancers15072067)

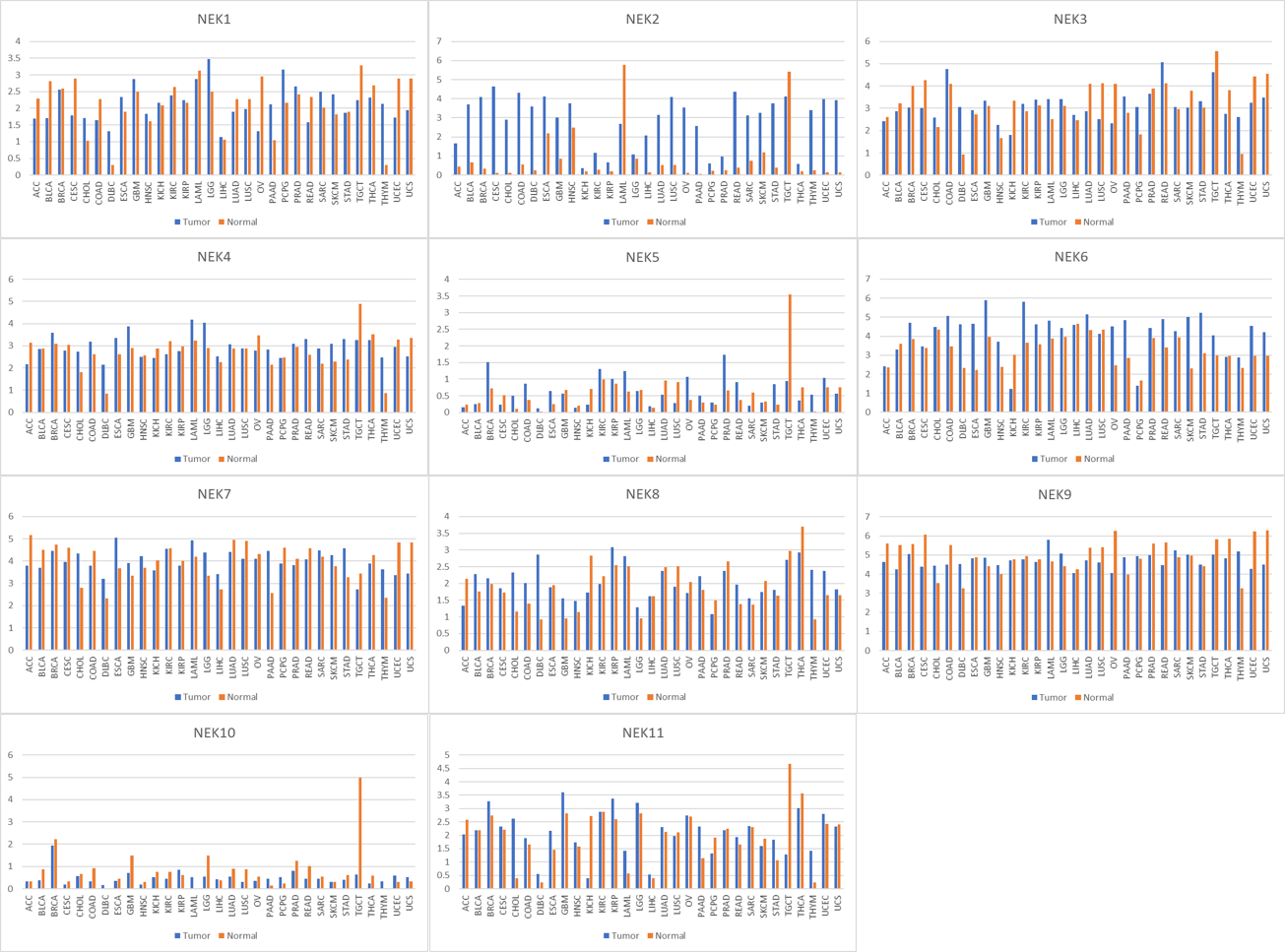

Supplement: Supplementary file 1 [file cancers-15-02067-s001.zip › Figure S1-GEPIA Analysis.png]

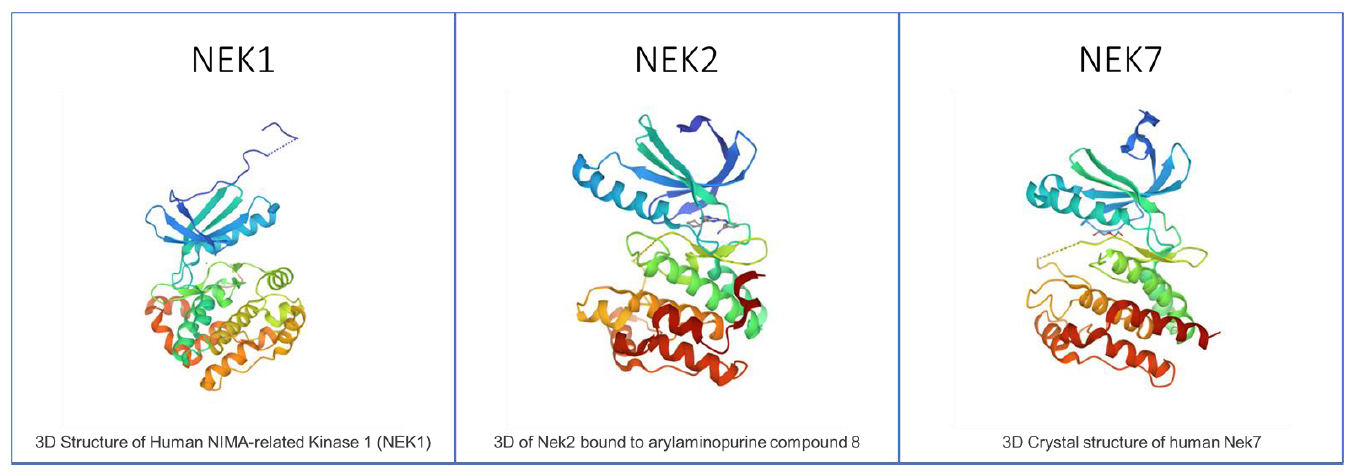

Supplement: Supplementary file 1 [file cancers-15-02067-s001.zip › Figure S2-3D Structure Models.png]
